# Berberin sustained-release nanoparticles were enriched in infarcted rat myocardium and resolved inflammation

**DOI:** 10.1186/s12951-023-01790-w

**Published:** 2023-01-28

**Authors:** Ke Zhu, Yu Yao, Kun Wang, Fuqiang Shao, Ziyang Zhu, Yangmeihui Song, Zhangyongxue Zhou, Dawei Jiang, Xiaoli Lan, Chunxia Qin

**Affiliations:** 1 Department of Nuclear Medicine, Union Hospital, Tongji Medical College, Huazhong University of Science and Technology, No. 1277 Jiefang Ave, Wuhan, 430022 Hubei China; 2 Hubei Key Laboratory of Molecular Imaging, Wuhan, 430022 Hubei China; 3 Department of Nuclear Medicine, The First People’s Hospital of Zigong, Zigong, Sichuan China; 4 Department of Ultrasound, The Central Hospital of Wuhan, Tongji Medical College, Huazhong University of Science and Technology, Wuhan, Hubei China; 5 Department of Nuclear Medicine, Shanghai East Hospital, Tongji University School of Medicine, Shanghai, China; 6 Key Laboratory of Biological Targeted Therapy, The Ministry of Education, Wuhan, 430022 Hubei China

**Keywords:** Myocardial infarction, Inflammation, Berberin, Nanoparticle, Targeted, Sustained release

## Abstract

Inflammatory regulation induced by macrophage polarization is essential for cardiac repair after myocardial infarction (MI). Berberin (BBR) is an isoquinoline tetrasystemic alkaloid extracted from plants. This study analyzes the most likely mechanism of BBR in MI treatment determined via network pharmacology, showing that BBR acts mainly through inflammatory responses. Because platelets (PLTs) can be enriched in the infarcted myocardium, PLT membrane-coated polylactic-co-glycolic acid (PLGA) nanoparticles (BBR@PLGA@PLT NPs) are used, which show enrichment in the infarcted myocardium to deliver BBR sustainably. Compared with PLGA nanoparticles, BBR@PLGA@PLT NPs are more enriched in the infarcted myocardium and exhibit less uptake in the liver. On day three after MI, BBR@PLGA@PLT NPs administration significantly increases the number of repaired macrophages and decreases the number of inflammatory macrophages and apoptotic cells in infarcted rat myocardium. On the 28th day after MI, the BBR@PLGA@PLT group exhibits a protective effect on cardiac function, reduced cardiac collagen deposition, improved scar tissue stiffness, and an excellent angiogenesis effect. In addition, BBR@PLGA@PLT group has no significant impact on major organs either histologically or enzymologically. In summary, the therapeutic effect of BBR@PLGA@PLT NPs on MI is presented in detail from the perspective of the resolution of inflammation, and a new solution for MI treatment is proposed.

**Graphical Abstract:**

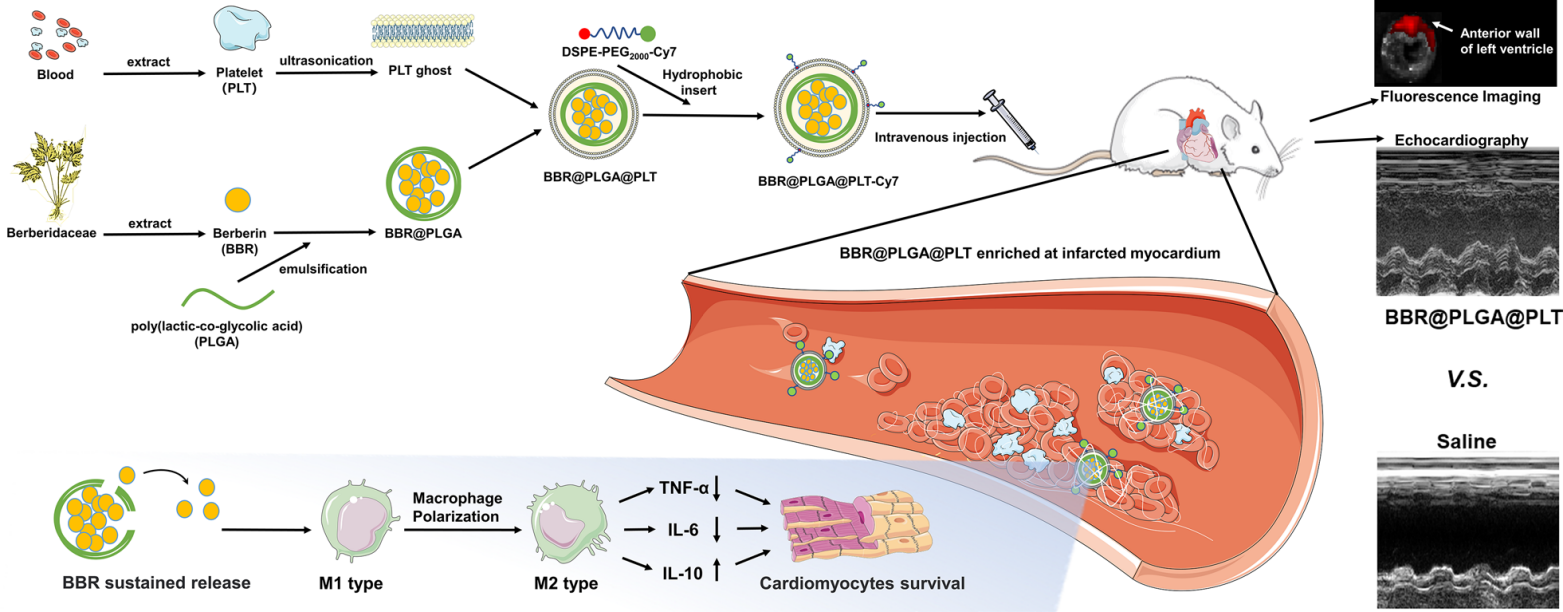

**Supplementary Information:**

The online version contains supplementary material available at 10.1186/s12951-023-01790-w.

## Introduction

Myocardial infarction (MI) and related complications comprise the leading cause of death in humans worldwide [1]. In addition to myocardial injury caused by ischemia and hypoxia, excessive local inflammation can aggravate myocardial injury after MI, which promotes cardiomyocyte apoptosis and accelerates the synthesis of extracellular matrix, leading to myocardial fibrosis, adverse ventricular remodeling, and cardiac dysfunction [2]. Early inflammatory activation in the infarcted myocardium is the basis for a later myocardial repair phase, but optimal repair after MI requires appropriate containment and resolution of inflammation [3]. Inflammatory macrophages (M1-type macrophages) enter the infarcted area at an early stage to secrete various cytokines, such as tumor necrosis factor (TNF)-α, leading to cardiomyocyte apoptosis. Several studies have shown that switching inflammatory macrophages to the reparative phenotype (M2-type macrophages) on time is essential for the containment and resolution of inflammation [4–7].

Berberin (BBR) and the genus Berberis have a long history in traditional medicine in China and other countries [8, 9]. Since 1975, BBR has been indexed in the Medical Subject Headings and has received wide attention from the scientific community. This natural product has anti-inflammatory, antioxidant, anti-diarrheal, antibacterial, cholesterol-lowering, and vasorelaxant effects [10, 11]. Notably, long-term oral administration of high doses of BBR has been reported to improve cardiac function and produce cardioprotective effects in mouse models of heart failure [9, 12, 13]. Therefore, BBR is a promising therapeutic agent to improve the cardiac function of post-MI patients. However, BBR has a low absorption rate in the intestines (less than 5%), and direct intravenous administration may produce side effects such as transient hypotension, which significantly limits its clinical use[14–16]. Therefore, it is necessary to develop new methods to resolve problems associated with the clinical application of BBR.

Cell membrane coating technology provides nanoplatforms with the ability to mimic source cells, including red blood cells, stem cells, bacteria, cancer cells, and platelets (PLTs) [17, 18]. PLT membrane is lipid bilayers embedded with glycoprotein integrins and has membrane integrin receptors such as GPIIb/IIIa, CD62P, immunomodulatory proteins, CD47, and CD55. These receptors can effectively help PLTs accumulation in inflammatory lesions, and improve the biocompatibility of PLT-coated nanoplatforms and prolong blood circulation through immune escape [19–22]. PLTs also possess the transmembrane proteins, and studies have shown that PLTs nanoplatforms can effectively bind to damaged regions of blood vessels via transmembrane proteins GPVI [23, 24].

The ability of PLTs to target inflammation, bind to damaged regions of blood vessels and their immune escape properties make PLT-like biomimetic carriers a promising option for delivering drugs to the infarcted myocardium. In addition, polylactic-co-glycolic acid (PLGA) is widely used for drug delivery because of its biodegradability and biocompatibility and is approved by the US Food and Drug Administration [25, 26]. Numerous studies have demonstrated that PLGA microspheres are highly suitable for use as sustained-release carriers [27–29].

**Scheme 1:**
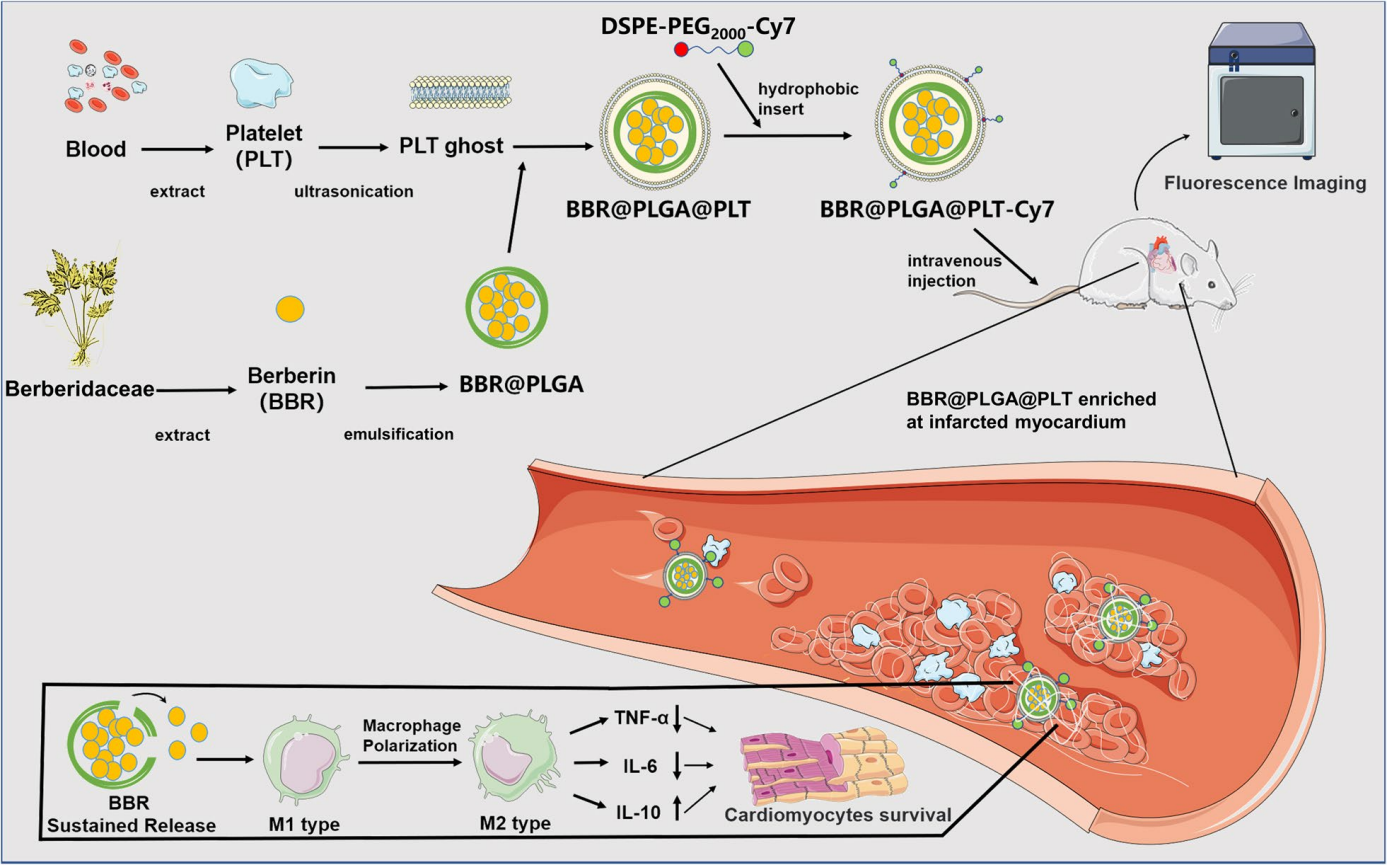
Schematic illustration of BBR@PLGA@PLT NPs for MI therapy. After BBR@PLGA@PLT NPs accumulate in infarcted myocardium via inflammation targeting, PLGA undergoes hydrolysis, and sustained release of BBR begins. A high concentration of BBR in infarcted myocardium promotes macrophage polarization to the M2 phenotype and protects cardiomyocytes

In this study, BBR was loaded in PLT membrane-coated PLGA nanoparticles (BBR@PLGA@PLT NPs) and administered intravenously (Scheme 1). Because of the characteristics of PLTs and PLGA mentioned above, sustained release of BBR was achieved in the infarcted myocardium. We also investigated the effect of BBR@PLGA@PLT on promoting M2 polarization in macrophages in the early MI phase and their ability to protect cardiac function within four weeks in rat models.

## Results and discussion

### Characterization of BBR@PLGA@PLT NPs

**Fig. 1:**
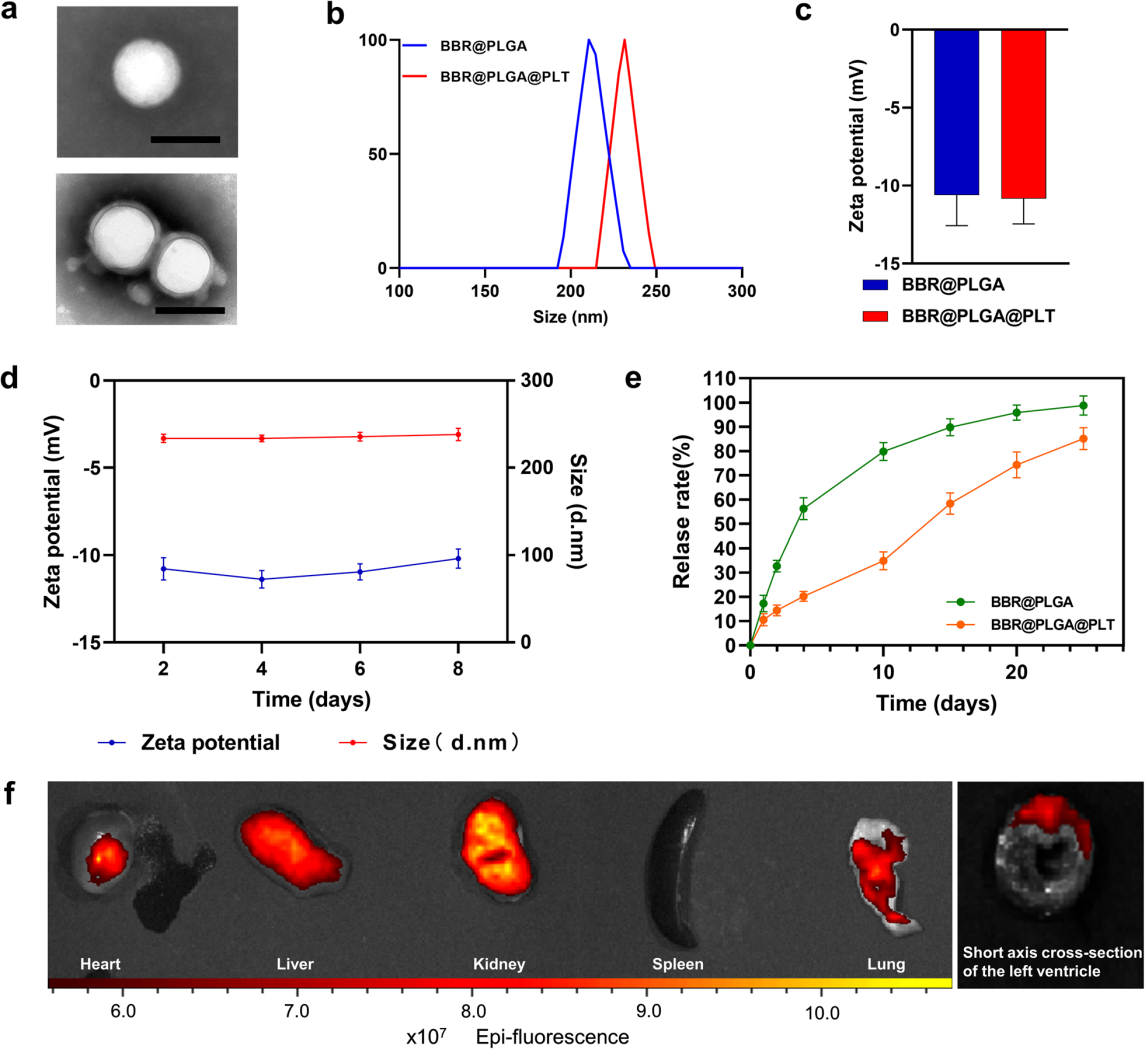
Characterization of the platelet (PLT) membrane-coated biomimetic nanoparticles and ex vivo fluorescence imaging of isolated major organs. **a** Transmission electron microscopy images of BBR@PLGA and BBR@PLGA@PLT NPs (scale bar = 200 nm). **b** The sizes and **c** zeta potentials of BBR@PLGA and BBR@PLGA@PLT NPs. **d** The trends of sizes and zeta potentials of BBR@PLGA@PLT NPs over time. **e** In vitro drug release profiles of BBR@PLGA and BBR@PLGA@PLT NPs. **f** Ex vivo fluorescence imaging of the heart and the other major organs (liver, kidney, spleen, and lung) of MI rats 24 h after DSPE-PEG_2000_-Cy7-labelled BBR@PLGA@PLT administration. All bars represent as means ± SD (n = 3)

The drug loading rate of BBR-loaded PLGA nanoparticles (BBR@PLGA NPs), as determined by liquid chromatography-mass spectrometry, was 10.07%, which confirmed that BBR@PLGA NPs were successfully prepared by a double emulsion process. As determined by transmission electron microscopy, BBR@PLGA NPs had a spherical morphology, with an average diameter of approximately 210 nm, BBR@PLGA@PLT NPs had a “core-shell” structured morphology (Fig. 1a), the single outer layer of the PLT “shell” was approximately 10 nm thick, consistent with the previously reported thickness of PLTs [30]. Dynamic light scattering analysis indicated that the peak hydrodynamic diameter of BBR@PLGA NP was 215 nm, with a polydispersity index of 0.161 and a zeta potential of − 10.8 mV (Fig. 1b, c). Compared with uncoated BBR@PLGA NP, the peak hydrodynamic diameter of BBR@PLGA@PLT NP was increased from 215 to 235 nm, which was attributed to the PLTs. Additionally, the absolute value of zeta potential of BBR@PLGA@PLT NP (− 11.4 mV) was higher than that of unmodified BBR@PLGA NP (Fig. 1b, c). Subsequently, the stability of BBR@PLGA@PLT NPs was tested, and no significant change was found in the zeta potential or nanoparticle size within eight days (Fig. 1d).

The in vitro release kinetics of BBR@PLGA and BBR@PLGA@PLT NPs are shown in Fig. 1e. PLT-coated nanoparticles had a smoother release curve. In the BBR@PLGA group, the drug was markedly released during the first four days of the experiment, and 79.83% ± 3.67% of BBR was released from day 0 to day 10. In contrast, drug release was relatively stable in the BBR@PLGA@PLT group, with BBR release of 34.90% ± 3.69% from day 0 to day 10. From the 10th to the 20th day, the drug release ability of the BBR@PLGA group was significantly decreased, with a drug release amount of 16.01% ± 1.04%. In contrast, in the BBR@PLGA@PLT group, 37.42% ± 0.68% of the drug was released. After 20 days of incubation, the BBR@PLGA and BBR@PLGA@PLT groups released 95.83% ± 3.09% and 74.32% ± 5.32% of the BBR, respectively. One of the disadvantages of PLGA nanoparticles is that initial drug burst release is common [31]. Both membrane-like nanoparticles and PLGA nanoparticles exhibit sustained drug release properties in vitro [32]. In the BBR@PLGA@PLT system, BBR@PLGA is coated by PLT membrane with a double sustained release effect, and the drug released from BBR@PLGA nanoparticles remains in the PLT membrane first and does not rapidly release outside of BBR@PLGA@PLT, which may be the reason why the drug release curve of BBR@PLGA@PLT is smoother than that of BBR@PLGA. This indicates PLTs could help in the delivery of drugs released early from BBR@PLGA nanoparticles to the infarcted myocardium, which may have a beneficial impact on treatment.

Distearoyl phosphatidylethanolamine (DSPE), as a hydrophobic group, can be inserted into the platelet membrane because of its excellent biological scalability. Therefore, DSPE-PEG_2000_-Cy7 was used to label BBR@PLGA@PLT fluorescently. Figure 1f shows the distribution of BBR@PLGA@PLT in the major organs of rats with acute MI 24 h after intravenous injection of nanoparticles. The fluorescence intensity in the infarcted myocardium is significantly higher than that in the non-infarcted area. As a control experiment, Cy7-encapsulated PLGA nanoparticles were administered to MI rats via the caudal vein. PLGA nanoparticles without platelet encapsulation had no immune evasion ability, so the liver captured most PLGA nanoparticles, and there was no difference in fluorescence intensity between the infarcted myocardium and the background (Additional file 1: Fig. S1).

The drug release kinetics and biodistribution results suggest that BBR@PLGA@PLT NPs have ability to remain in circulation for a long time and reduce the potential to be trapped in the liver, which allows more nanoparticles to be enriched in the infarcted myocardium. The PLT coating technology provides BBR@PLGA NPs with the ability to escape the immune response and become enriched in damaged vessels and inflammatory lesions [33]. In addition, PLTs have good biological expansibility and can be further modified. BBR@PLGA@PLT NPs can facilitate the targeted delivery of anti-inflammatory drugs such as BBR into infarcted myocardium.

### Network pharmacologic analysis of the effect of BBR on MI

**Fig. 2:**
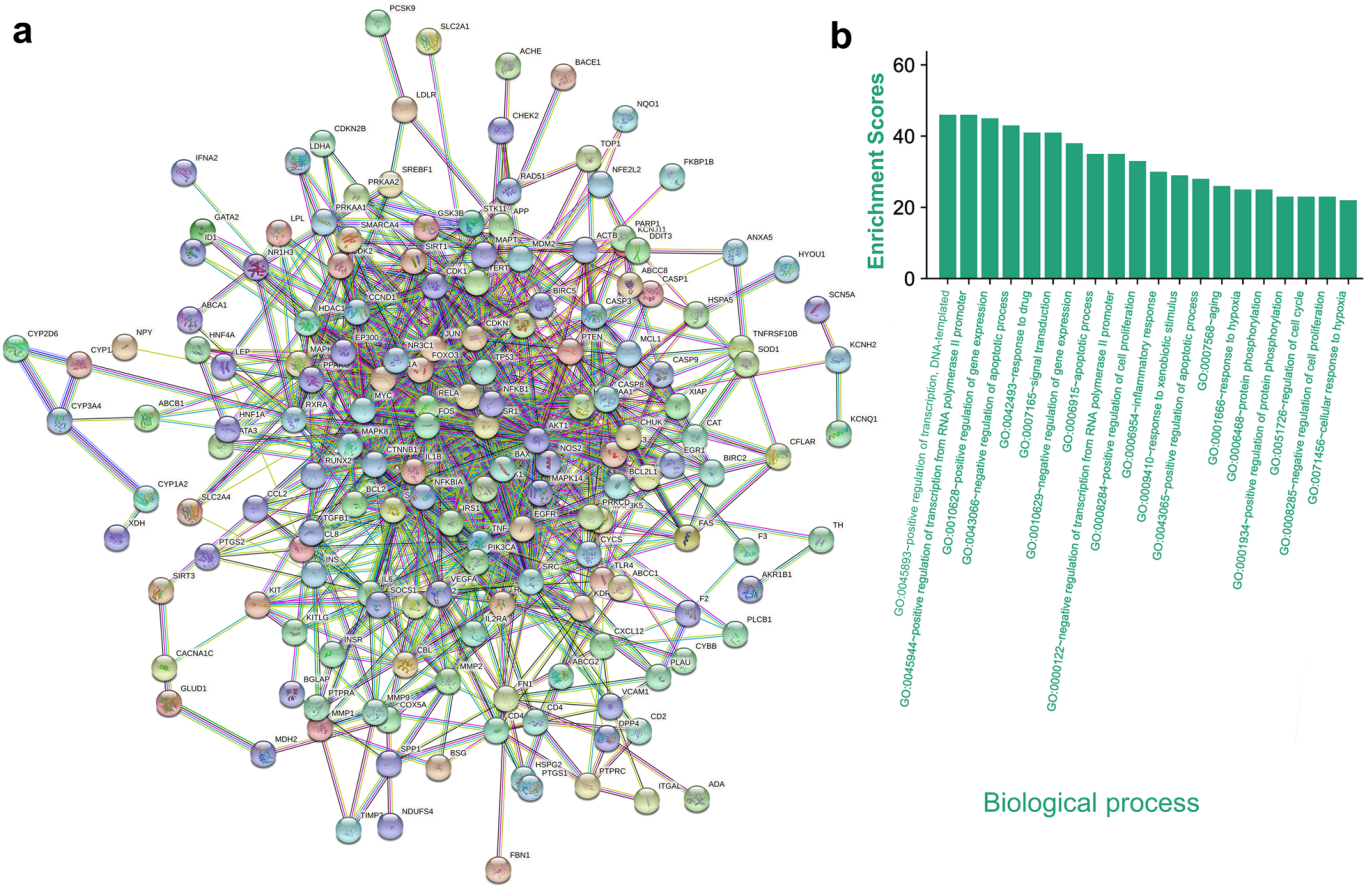
Interaction network of the overlapping targets. **a** Protein–protein interaction network of the overlapping targets. **b** Gene Ontology enrichment analysis of the overlapping targets

In traditional Chinese medicine, because the therapeutic mechanism of natural drugs is extremely complex, compounds composed of natural drugs are often used to treat patients through network interactions [34]. The most important mechanism of BBR in the treatment of MI is difficult to confirm by quantitative analysis in animal experiments. Network pharmacology can be used to predict the mechanism, toxicity, and metabolic characteristics of traditional Chinese medicines [35]. Therefore, we conducted a network pharmacological analysis of the mechanism of BBR action in acute MI. To determine their interactions, 289 target genes associated with BBR and 1837 target genes associated with acute MI were analyzed. A protein-protein interaction network was constructed using 200 different genes considered key targets in acute MI treatment (Fig. 2a), which indicated that the core mechanisms of BBR effect on MI involve centrally located interleukin (IL)-1β, TNF, and nuclear factor kappa-B, etc.

Gene Ontology enrichment analysis was performed to reveal the biological characteristics of 200 intersecting target genes. Detailed network pharmacology data are provided in Additional file 1: Fig. S2–S4. The top 20 terms significantly enriched for biological processes (Fig. 2b) included negative regulation of the apoptotic process, positive regulation of the apoptotic process, positive regulation of cell proliferation, inflammatory response, and response to hypoxia, which were all closely associated with the treatment of MI. Therefore, we speculated that BBR exerted a protective effect on the myocardium through the above biological processes. Because the effects of natural drugs are often multifaceted, we excluded terms that were in conflict and those appeared as outcomes, we concluded that the therapeutic effect of BBR on MI mainly involves the inflammatory response and hypoxia response. The enrichment score of the inflammatory response was much higher than that of the hypoxia response; therefore, we primarily verified the effect of BBR@PLGA@PLT NPs on the inflammatory response.

### Ability of BBR@PLGA@PLT NPs to regulate inflammation in vitro and in vivo

**Fig. 3:**
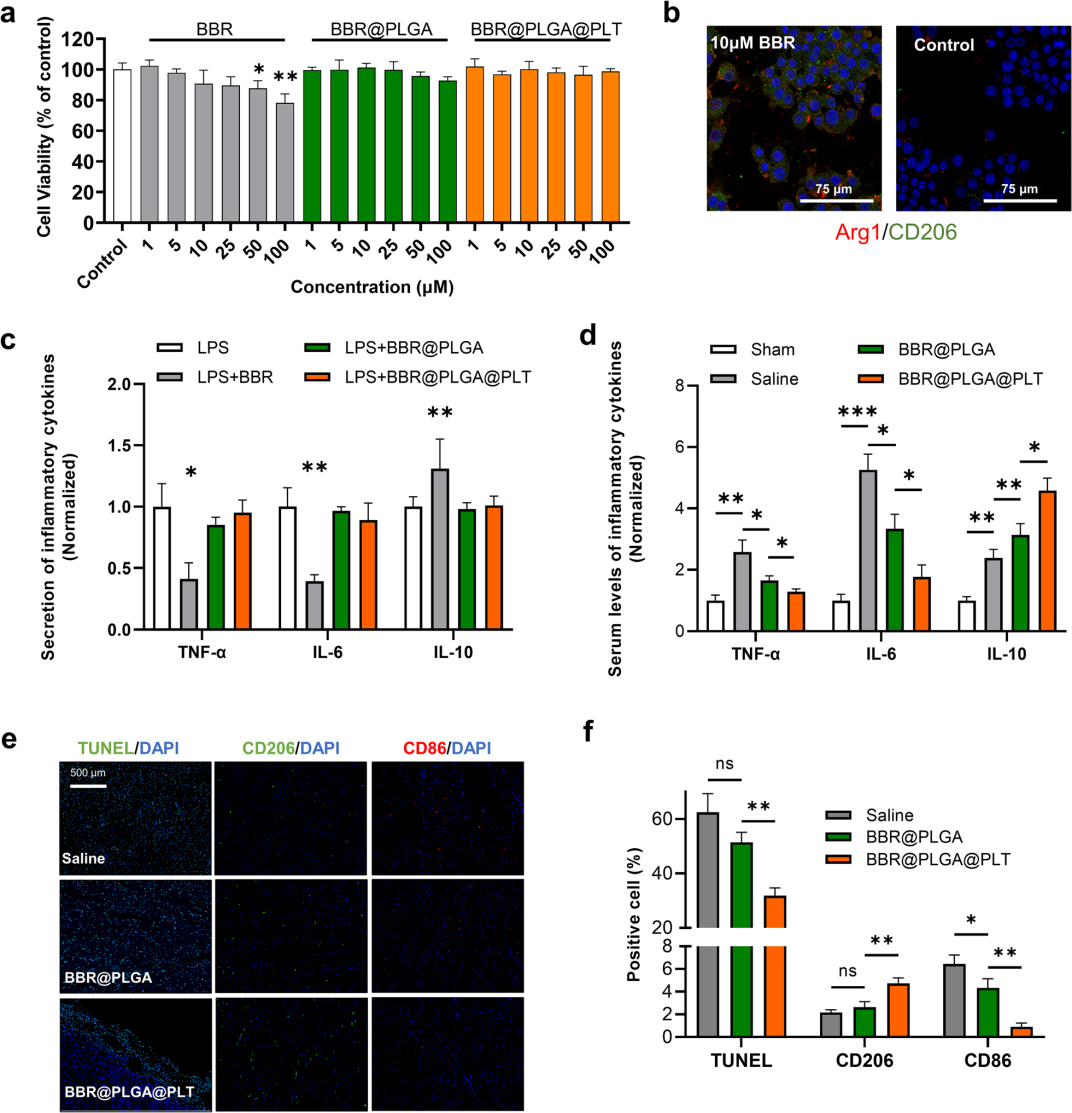
The in vitro and in vivo effects of BBR@PLGA@PLT NPs on macrophages. **a** Cell viability of macrophages after incubation with various concentrations of BBR, BBR@PLGA, and BBR@PLGA@PLT NPs in solution for 12 h. **b** Confocal microscopic images of macrophage markers after incubation in 10 μM BBR solution and saline for 12 h. **c** Inflammatory cytokines secreted by RAW 264.7 cells. **d** Serum levels of inflammatory cytokines in rats on day three after MI. **e** Representative immunofluorescence images of TUNEL, CD206, and CD86 in the border zone of infarcted hearts on day three after MI. TUNEL-stained (green) cells indicate apoptosis-positive cells, and CD86 (red) and CD206 (green) staining shows M1 and M2-type macrophages, respectively. **f** Statistical analysis of the percentage of TUNEL-, CD206-, and CD86-positive cells. All bars represent as means ± SD (n = 3). *P < 0.05 and **P < 0.01, ***P < 0.001

Subsequently, we evaluated the in vitro biological effects of BBR@PLGA@PLT NPs. The cytotoxicity of BBR@PLGA@PLT NPs was investigated in macrophages (Fig. 3a). In both the BBR@PLGA group and BBR@PLGA@PLT group, BBR was coated with nanoparticles and entered the macrophage environment in a sustained release manner. Therefore, the BBR concentration in the cell environment was relatively low in the BBR@PLGA and BBR@PLGA@PLT groups compared with that in the BBR aqueous solution group. The cell viability decreased only slightly with increasing BBR concentration. Only the BBR aqueous solution groups that contained 50 μg/mL and 100 μg/mL of BBR showed significant reductions in the viability of RAW 264.7 cells (*P* = 0.0304, *P* = 0.0062). These results demonstrated that the sustained release ability of BBR@PLGA and BBR@PLGA@PLT NPs reduced the toxicity of BBR and that PLGA and PLTs are biocompatible.

As shown in Fig. 3b, the BBR aqueous solution group showed significant up-regulation of ARG1 and CD206 (M2 subtype marker) in RAW 264.7 cells compared with the saline control group. We demonstrated that 10 μM BBR did not affect macrophage viability (Fig. 3a), suggesting that BBR exerts its effect on macrophages mainly by acting on macrophage M2-subtype polarization rather than directly inhibiting macrophage activity.

The normalized levels of inflammatory cytokines secreted by RAW 264.7 cells are shown in Fig. 3c (the non-normalized data are shown in Additional file 1: Fig. S5). Only the BBR solution group showed significant differences, with down-regulation of IL-6 (a proinflammatory factor) and TNF-α (a major cytokine that mediates cardiomyocyte apoptosis by inflammation) secretion and up-regulation of IL-10 (an anti-inflammatory factor) secretion. These data again demonstrated that BBR was released from BBR@PLGA and BBR@PLGA@PLT NPs in a sustained manner, and PLGA and PLTs did not produce changes in macrophage secretion in vitro.

In vivo experiments, the serum levels of IL-10, IL-6, and TNF-α were increased on day four after MI, and the IL-10 level was further increased after BBR@PLGA and BBR@PLGA@PLT treatment, while the TNF-α and IL-6 levels were decreased. Moreover, the BBR@PLGA@PLT group had the highest serum IL-10 level and the lowest IL-6 and TNF levels (all *P* < 0.05) (Fig. 3d, the non-normalized data are shown in Additional file 1: Fig. S5).

Thus, we reasoned that the most likely mechanism of action of BBR@PLGA@PLT NPs on MI involved the cardiac enrichment capacity of the PLT coating, the sustained BBR release capacity of PLGA nanoparticles, and the ability of BBR to promote M2 polarization in macrophages.

We demonstrated that BBR@PLGA@PLT NPs could promote M2 polarization of macrophages in infarcted myocardium by measuring the expression of markers, including TUNEL (apoptotic marker), CD206 (M2 macrophage surface marker), and CD86 (M1 macrophage surface marker) on day four post-MI (Fig. 3e). There was no significant difference in the percentage of TUNEL-positive cells (apoptotic cardiomyocytes) and CD206-positive cells between the saline group and BBR@PLGA group (*P* = 0.0683, *P* = 0.2088). The only significant difference between these groups was in the percentage of CD86-positive cells (*P* = 0.0328). The BBR@PLGA@PLT group showed significant differences in all markers compared with the BBR@PLGA group. Apoptotic cardiomyocytes (*P* = 0.0018) and CD86-positive cells (*P* = 0.0025) were significantly reduced, and CD206-positive cells were increased significantly in the BBR@PLGA@PLT group compared with the BBR@PLGA group (*P* = 0.0062) (Fig. 3f).

### Evaluation of cardiac function by echocardiography

**Fig. 4:**
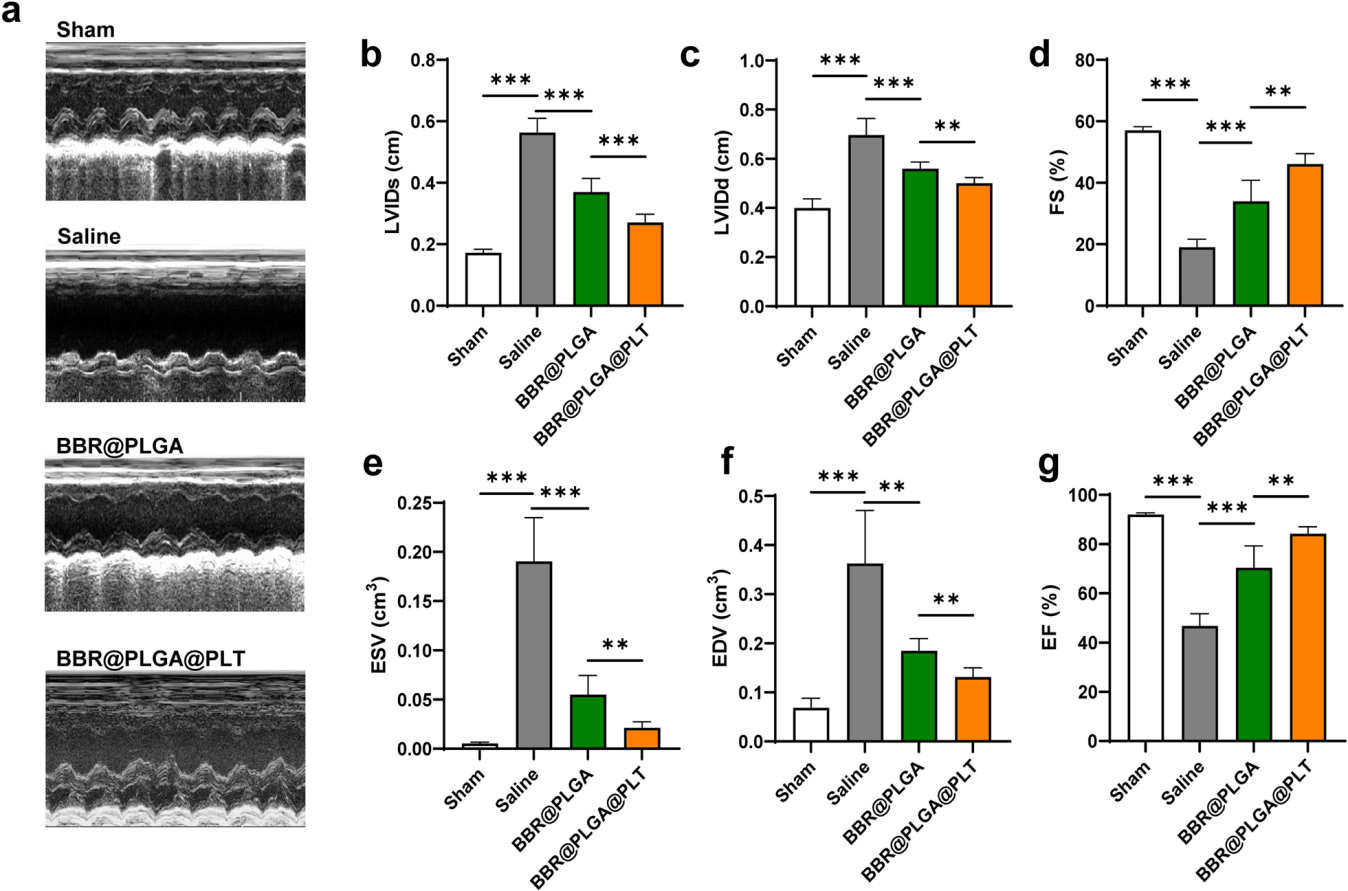
Cardiac function was evaluated by echocardiography on day 28 after MI. **a** Representative echocardiography images of different groups. **b** LVIDs. **c** LVIDd. **d** FS. **e** ESV. **f** EDV. **g** LVEF. All bars represent as means ± SD (n = 6). *P < 0.05 and **P < 0.01, ***P < 0.001

Cardiac function is a direct response to the therapeutic effect of drugs on MI, and was measured by echocardiography in rat models on day 28 after MI. Representative echocardiograms are shown in Fig. 4a. Compared with the sham group, the MI rats treated with saline exhibited typical characteristics of heart failure with a significantly increased left ventricular internal diameter at end-diastole (LVIDd), left ventricular inner diameter at end-systole (LVIDs), end-systolic volume (ESV), and end-diastolic volume (EDV), as well as reductions in left ventricle fractional shortening (FS) and left ventricular ejection fraction (LVEF) (all *P* < 0.05) (Fig. 4b–g). BBR@PLGA NPs demonstrated a limited protective effect on heart function with increased EF and FS and decreased in LVIDd, LVIDs, EDV, and ESV compared with saline (all *P* < 0.05). The BBR@PLGA@PLT group had the highest EF and FS and the lowest LVIDd, LVIDs, EDV, and ESV among all MI groups (all *P* < 0.05) (Fig. 4b–g).

### Evaluation of fibrosis by pathological staining and western blotting

**Fig. 5:**
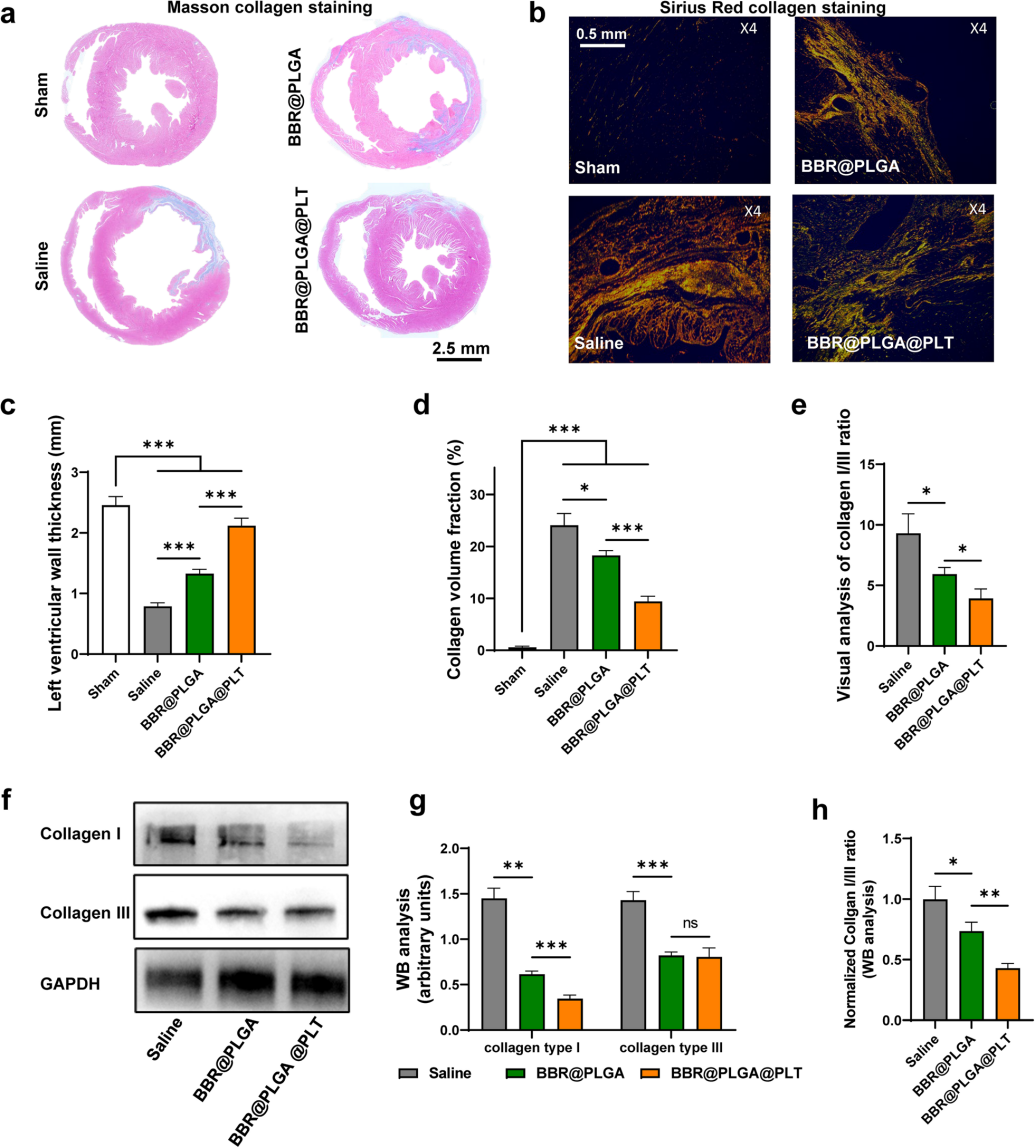
Evaluation and quantitative analysis of fibrosis by pathological staining and western blotting on day 28 after MI. **a** Representative images of heart sections stained with Masson trichrome, collagen is stained blue. **b** Representative images of heart sections stained with Sirius Red, the red and yellow tissues are considered type I collagen, while the green tissues are type III collagen. Quantitative analysis of the **c** ventricular wall thickness, **d** collagen content, and **e** collagen I/III ratio in different groups was performed by visual analysis. **f** Western blot results, **g** quantification of collagen content, and **h** collagen I/III ratio. All bars represent as means ± SD (n = 3). *P < 0.05 and **P < 0.01, ***P < 0.001

Myocardial fibrosis is an important pathological process that leads to adverse cardiac remodeling and fatal heart failure [36, 37]. The pathological manifestation of fibrosis is that excess collagen deposition replaces normal tissue. Systematic assessment of myocardial fibrosis on day 28 showed that BBR@PLGA@PLT NPs reduced collagen accumulation and improved collagen composition (Fig. 5).

The saline group had a large amount of collagen deposited in the ventricle, which was stained blue by Masson staining (Fig. 5a). Stiff collagen lacks the contractile capacity of cardiomyocytes and reduces ventricular thickness during the long-term passive stretch (Fig. 5c). Compared with the saline group, the BBR@PLGA group had greater ventricular thickness and less collagen deposition. The ventricles of the BBR@PLGA@PLT group had the thickest ventricular wall and the least collagen deposition among all MI groups (Fig. 5d).

The collagen fibers observed by Masson staining were composed of multiple components. Type III collagen has good elasticity, and type I collagen is stiff [37]. The collagen I/III ratio can reflect the passive expansion ability of collagen fibers in scar tissue. In this study, type I and type III collagen were observed using Sirius Red staining. The visual analysis results showed that the BBR@PLGA group had a lower collagen I/III ratio than the saline group (*P* = 0.0267), and the BBR@PLGA@PLT group had a significantly lower collagen I/III ratio than the BBR@PLGA group (*P* = 0.022) (Fig. 5e). To ensure the reliability of this conclusion, we also performed western blotting analysis of collagen in the infarcted myocardium (Fig. 5f). The densitometry results are presented as arbitrary units and normalized ratios. Semi-quantitative analysis showed that treatment with BBR@PLGA and BBR@PLGA@PLT NPs significantly reduced the content of type I and type III collagen in the scar tissue on day 28 (all *P* < 0.05). The BBR@PLGA@PLT group exhibited a greater reduction in type I collagen than the BBR@PLGA group (*P* = 0.0003), but there was no significant difference in the type III collagen content between the two groups (*P* = 0.8078) (Fig. 5g). The BBR@PLGA@PLT group had the lowest type I/III collagen ratio, which was significantly lower than that in the BBR@PLGA group (*P* = 0.0031) (Fig. 5h). Thus, we again verified the effect of BBR@PLGA@PLT NPs on improving the composition of scar tissue by western blot analysis. The elasticity of collagen fibers in scar tissue plays an important role in the protection of cardiac function.

Fibroblasts secrete collagen in the myocardium and other organs. Studies [37, 38] have shown that the secretion of collagen by fibroblasts in infarcted myocardium is mainly regulated by inflammatory factors, such as IL-10. IL-10 increases collagen synthesis in the early phase of MI, but inhibits collagen synthesis and regulates collagen composition in the later phase of MI, leading to the up-regulation of type I collagen in scar tissue. Type I collagen, which has greater elasticity than type III collagen, can reduce fibrotic myocardial stiffness and directly affect cardiac function. Importantly, cardiac stiffness is one of the leading causes of decompensation in heart failure [39]. Therefore, we suggest that BBR@PLGA@PLT NPs can be enriched in the infarcted myocardium, and the released BBR can regulate the secretory phenotype of macrophages to reduce fibrosis of the infarcted myocardium and stiffness of cardiac scar tissue.

### Assessment of cardiac structures

Cardiac structures, including electrical signal transmission networks, vascular networks, and cardiomyocytes [40], were assessed on day 28 by immunofluorescence analysis.

**Fig. 6:**
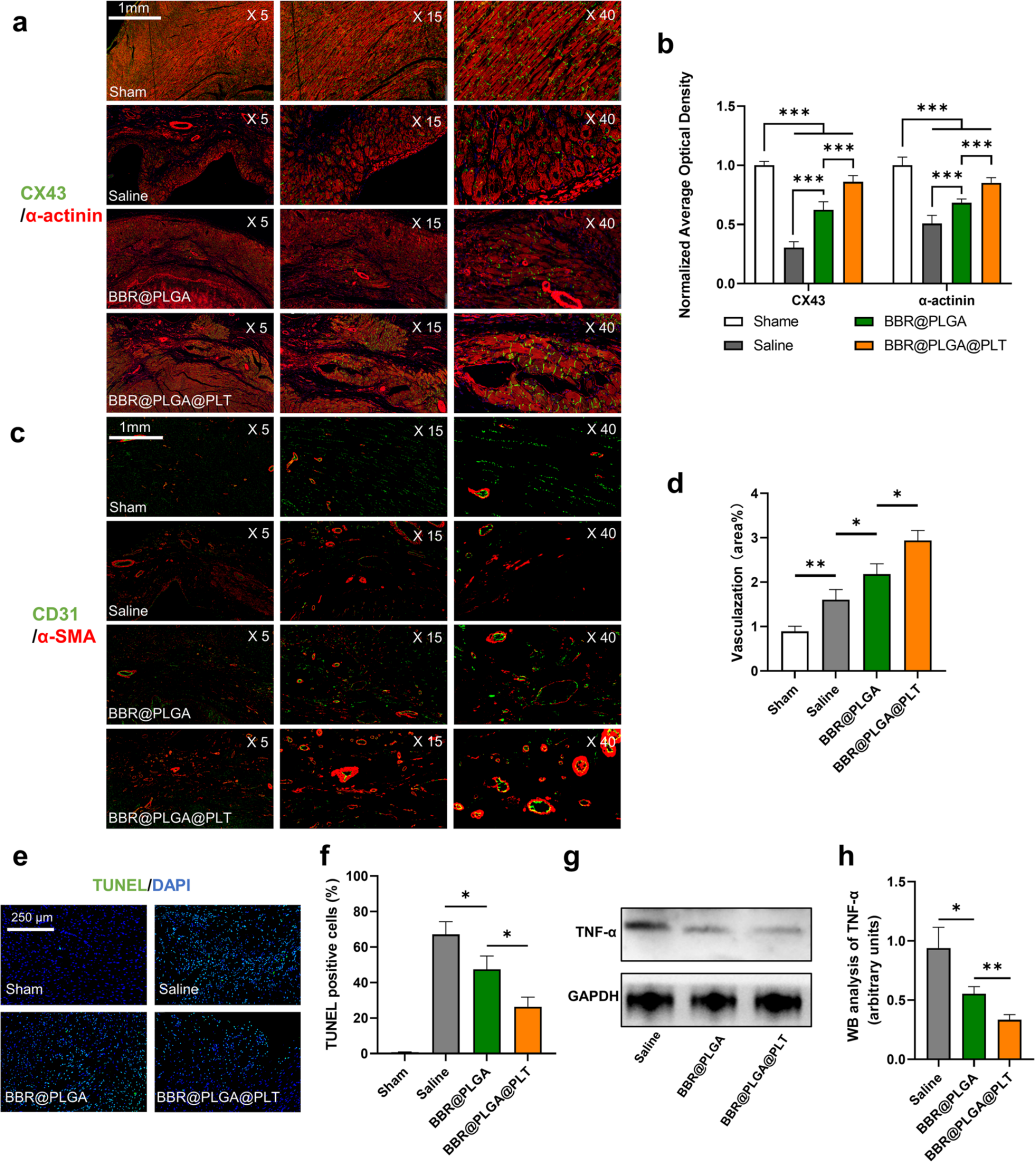
Assessment of cardiac structures, including electrical signal transmission networks, vascular networks, and cardiomyocytes on day 28 after MI. **a** Representative immunofluorescence images co-stained for CX43 (green) and α-actinin (red). **b** Optical density semi-quantitative analysis of CX43 and α-actinin expression. **c** Representative immunofluorescence images of blood vessels co-stained for CD31 (green) and α-SMA (red). **d** Quantitative analysis of the neovessels in the infarct regions. **e** Evaluation of cardiomyocyte apoptosis in the border zone of infarcted hearts by TUNEL staining and **f** semi-quantitative analysis. **g** Western blot and **h** quantification of TNF-α expression in infarcted myocardium. All bars represent as means ± SD (n = 3). *P < 0.05 and **P < 0.01, ***P < 0.001

Myocardial structure damage was detected by measuring α-actinin and CX43 protein levels. α-actinin is a vital protein of the myocardial skeleton, and CX43 mediates electrical signal transmission between cardiomyocytes. As shown in Fig. 6a, MI operation significantly changed the myocardium structure. CX43 and α-actinin were significantly down-regulated in the infarcted myocardium of the saline group. Additionally, the location of CX43 in the tissue was altered; CX43 was absent from viable myocardial tissue and mainly distributed around the nuclei of cardiomyocytes. Compared with the saline group, both the BBR@PLGA and BBR@PLGA@PLT groups had CX43 in the correct location. Furthermore, CX43 and α-actinin protein levels were up-regulated in the BBR@PLGA and BBR@PLGA@PLT groups, with greater up-regulation in the BBR@PLGA@PLT group than that in the BBR@PLGA group (all *P* < 0.0001) (Fig. 6b).

In infarcted myocardium, cardiomyocytes are more likely to survive when angiogenesis occurs [41]. As shown in Fig. 6c, d, compared with the sham group, the saline group showed angiogenesis induced by MI operation. The vascular area of the infarcted myocardium in the saline group was significantly greater than that of the normal myocardium in the sham group (*P* = 0.0088). The BBR@PLGA group exhibited more vascularization than the saline group (*P* = 0.0391), and the angiogenesis of infarcted myocardium in the BBR@PLGA@PLT group was significantly greater than that in the BBR@PLGA group (*P* = 0.0162).

As shown in Fig. 6e, f, compared with the sham group, all MI groups showed obvious cardiomyocyte apoptosis. The number of apoptotic cells in the BBR@PLGA group was significantly lower than that in the saline group (*P* = 0.0304), while the number of apoptotic cells in the BBR@PLGA@PLT group was lower than that in the BBR@PLGA group (*P* = 0.0169). TNF-α is produced predominantly by macrophages, and it is a major cytokine responsible for cardiomyocyte apoptosis [42]. Western blotting was performed to evaluate the TNF-α content in infarcted myocardium (Fig. 6g). The BBR@PLGA@PLT group had the least TNF-α in the infarcted myocardium (all *P* < 0.05), consistent with TUNEL staining.

BBR@PLGA@PLT NPs can protect the infarcted myocardium through various mechanisms by regulating inflammatory responses, such as down-regulation of TNF-α secreted by macrophages directly involved in reducing cardiomyocyte apoptosis [43]. Moreover, M1-type macrophages have been suggested to mainly promote vessel sprouting, while M2-type macrophages mainly promote the maturation and quiescence of new blood vessels. M1-type macrophages can cause degeneration of previously formed vessels, suggesting that promoting polarization of inflammatory macrophages to the M2 subtype after MI is critical for angiogenesis [4–7]. The BBR@PLGA@PLT group formed more mature arterioles consisting of CD31-positive cells (endotheliocytes) surrounded by α-smooth muscle actin (α-SMA)-positive cells (smooth muscle cells), demonstrating the ability of BBR@PLGA@PLT NPs to protect cardiomyocytes by resolving inflammation and up-regulating angiogenesis.

### Biosafety assessment

**Fig. 7:**
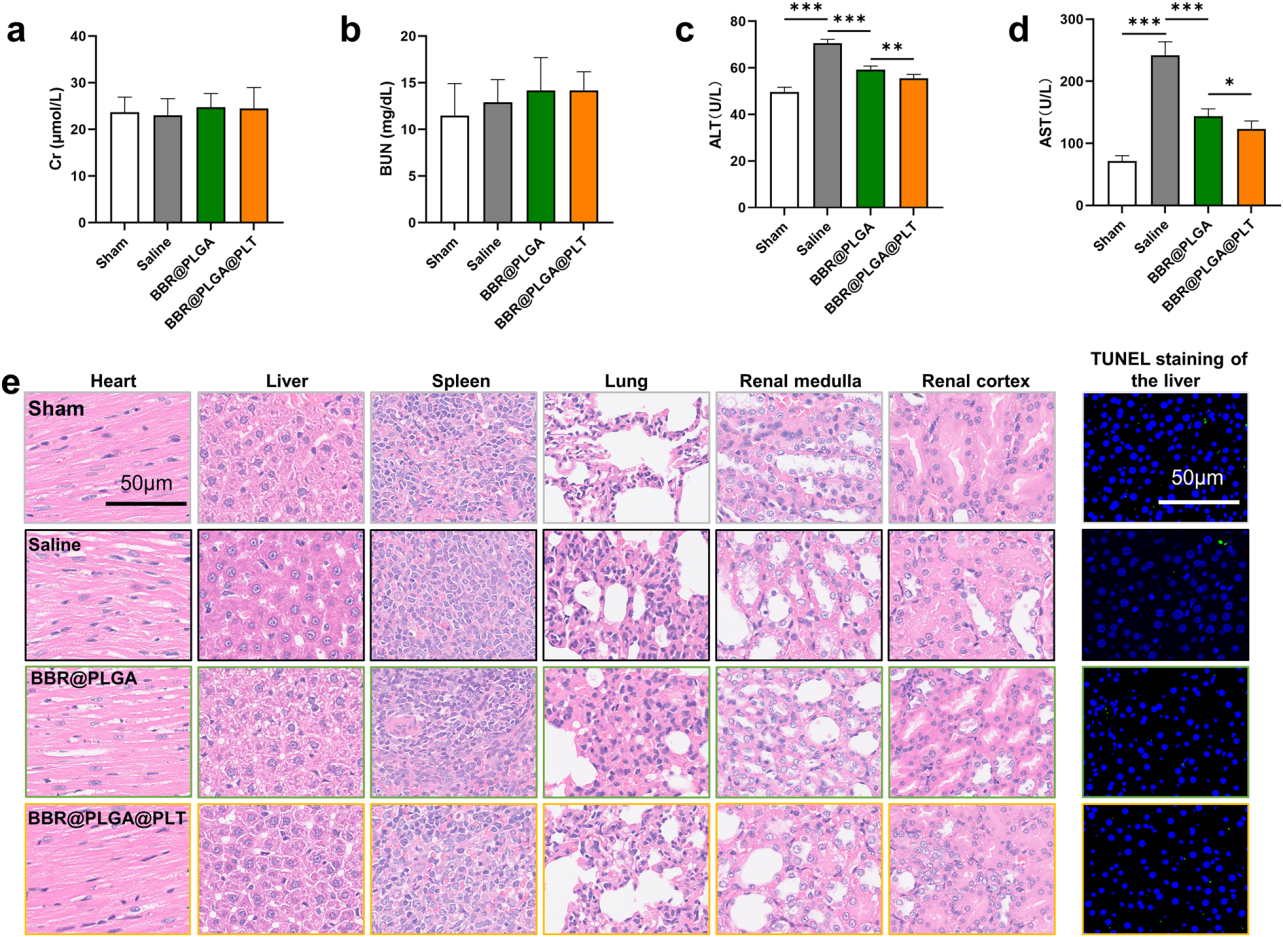
Preliminary safety evaluation. **a**–**d** Biochemical markers relevant to hepatic and kidney function. **e** Hematoxylin and eosin staining of sections of major organs resected from mice and subjected to treatment with various formulations for one month. All bars represent as means ± SD (n = 6)

The toxic side effects of nanoparticles on major organs and whole systems are a major concern during drug treatment. Potential side effects were investigated to assess biosafety in vivo. Biochemical analysis showed no significant differences in creatinine (Cr) and blood urea nitrogen (BUN) levels between any two groups, indicating that renal function was not affected by BBR@PLGA@PLT NPs treatment (Fig. 7a, b).

The serum alanine aminotransferase (ALT) and aspartate aminotransferase (AST) levels are commonly used to assess the toxicity of a drug to the liver and heart, respectively [44]. Compared with the saline group, the serum ALT level decreased after BBR@PLGA and BBR@PLGA@PLT NPs treatment (*P* < 0.0001), and the decrease was more obvious in the BBR@PLGA@PLT group (Fig. 7c), indicating that both BBR@PLGA and BBR@PLGA@PLT NPs had a protective effect on MI in rats, but the effect of BBR@PLGA@PLT NPs was superior to that of BBR@PLGA NPs. The BBR@PLGA@PLT group had the lowest serum ALT level among all MI groups (*P* < 0.0001, *P* = 0.0025) (Fig. 7d). However, in a comparison of the sham and saline groups, we concluded that MI operation resulted in increased serum AST and ALT levels in rats on day 28 (all *P* < 0.0001). Myocardial injury also causes an increase in the serum AST level; therefore, it is difficult to evaluate whether the drug injures the liver or has a protective effect via the serum AST level. Consequently, we performed a histological evaluation of the major organs (Fig. 7e). Hematoxylin and eosin staining showed no obvious histologic damage in the hearts, livers, spleens, lungs, or kidneys isolated from the BBR@PLGA@PLT group. TUNEL staining on liver sections showed that hepatocytes in each group exhibited no obvious apoptosis. All of the above results indicated that no obvious side effects occurred in other major organs.

## Conclusions

We successfully developed a BBR sustained-release nanomedicine that can be enriched in infarcted myocardium and used for MI therapy. We examined the mechanism of BBR in MI treatment by network pharmacology analysis and demonstrated that BBR@PLGA@PLT NPs could protect cardiac function, reduce adverse remodeling of the heart, maintain the structural integrity of myocardial tissue, protect cardiomyocytes, promote angiogenesis of infarcted myocardium by resolution of inflammation in MI model rats. This study indicates a new direction for MI treatment.

## Materials and methods

All animal experimental protocols were approved by the laboratory animal center of Tongji Medical College, Huazhong University of Science and Technology. All animal experiments were carried out according to the principles of the Declaration of Helsinki. All the chemicals used in this study that were not explicitly described were of analytical grade and were obtained from Sinopharm Group Chemical Reagent Company (Shanghai, China).

### Platelet and RBC membrane preparation

Fresh whole blood was collected from 300 ± 20 g male Sprague Dawley (SD) rats from postcava and then centrifuged at 250 g for 15 min at room temperature to differentiate the PLT-rich plasma supernatant from red blood cells and white blood cells in the bottom and middle layers. To prevent platelet activation, phosphate buffer saline (PBS) containing one mM ethylenediamine tetraacetic acid (EDTA, Solarbio, Beijing, China) and two mM Prostaglandin E1 (PGE1, Solarbio, Beijing, China) was added. The PLT-rich plasma was centrifuged again at 150 g for 25 min to remove the remaining blood cells. After centrifugation at 700 g at room temperature for 25 min, platelets were collected. The PLT extracted from 10 mL of blood was mixed with 2 mL of PBS containing one mM EDTA and protease inhibitor tablets to prevent protease activation. To extract the PLT membrane, PLTs were frozen at − 80 °C for two h, thawed at 37 °C, centrifuged at 3800* g* for 3 min, and resuspended in EDTA and protease inhibitor tablets containing PBS (3 mL). After repeated freezing and thawing three times, the platelet ghosts were obtained.

### Fabrication of BBR@PLGA@PLT NPs

BBR@PLGA NPs were fabricated by the W1/O/W2 double-emulsion technique. 10 mL of dichloromethane was used to dissolve 500 mg of PLGA (lactide/glycolide ratio of 75:25, Mw: 4 k–15 k Da, Macklin, Shanghai, China), and 2 mL of freshly prepared Berberin (BBR, Yuanye, Shanghai, China) solution (the ratios were 100 mg BBR, 300μL DMSO, 4.7 mL ultrapure water) was added to the PLGA solution. Then, a probe sonicator (LC1000N, Cheng Yue, Shanghai) was used to sonicate the mixture in an ice bath for 500 s, alternating on and off at 10% amplitude for 5 min. The mixture (W1/O) was rapidly added to 25 mL of 1% PVA1788 (Macklin, Shanghai, China) solution and homogenized at high speed for 10 min in an ice bath (W1/O/W2) at 12000 revolutions per min. The suspension was added to 30 mL of 3% isopropanol (Solarbio, Beijing, China)) solution and stirred with a magnetic stirrer at 25 °C overnight to remove the remaining dichloromethane. BBR@PLGA was washed three times by centrifugation with deionized water (12000 g, 20 min). Lastly, the BBR@PLGA was lyophilized for 48 h and packed into a sealed penicillin bottle. Dispersions of platelets ghosts were sonicated in a bath sonicator at 53 kHz and 100 W output power for 3 min to accomplish the cell membrane coating. 2 mL of deionized water containing 5 mg of respirable particles was mixed with PLT ghosts from 6 ml of whole blood.

### Characterization of BBR@PLGA@PLT NPs

Zeta PALS (Brookhaven Instruments, USA) was used to determine the average diameters, polydispersity index, and zeta potentials of BBR@PLGA and BBR@PLGA@PLT NPs; the measurement was made at 25 ℃, the laser wavelength was 660 nm, and all measurements were repeated for three times. At 2, 4, 6, and 8 days after preparation, diameter changes of BBR@PLGA and BBR@PLGA@PLT NPs were measured again. The prepared nanoparticles were all stored at 4 °C. Samples were stained negatively with 1% phosphotungstic acid for TEM. To determine the accumulation of BBR@PLGA and BBR@PLGA @PLT NPs in the heart, we injected DSPE-PEG_2000_-Cy7 (Xi'an Rui Xi Biotechnology Co Ltd, Xi'an, China) labeled BBR@PLGA @PLT NPs or Cy7-labeled BBR@PLGA NPs into MI rats via tail vein, the heart and major organs, like liver, kidney, spleen, and lung, were harvested 24 h after injection and imaged ex vivo by IVIS^®^ Spectrum In Vivo Imaging System (PerkinElmer).

### Network pharmacologic analysis of the effect of BBR on acute MI

GeneCards (http://www.genecards.org), Online Mendelian Inheritance in Man (http://omim.org/), and DisGeNET database (http://www.disgenet.org/web/DisGeNET/) were searched with the keywords "acute myocardial infarction" to identify acute MI-related genes. Using the online tool Venny 2.1 (http://bioinfogp.cnb.csic.es/tools), a Venn diagram was generated to identify the genes that interact with the target genes of Berberin. The interaction genes between Berberin and acute MI were regarded as the core genes and were analyzed to build the PPI network using the STRING online tool (https://string-db.org), where the confidence score > 0.9, the species was “Homo sapiens”. Based on the TSV format file from the STRING database, the key topological parameters, such as degree and betweenness centrality, were demonstrated through CytoNCA plug-in in Cytoscape v3.7.2, and the top 20 nodes were selected as core targets according to the degree value. GO enrichment analysis was conducted by importing the overlapping genes into Metascape (https://metascape.org/). The enrichment terms with *p* < 0.05 were collected, and those with *p* < 0.01 were considered as the critical value of significant pathways and functions. Finally, the top 20 biological processes (BP), cellular components (CC), and molecular functions (MF) were defined as terms with *p* < 0.01, and the pathways were identified based on *p* < 0.05. Then, the drug-target-pathway (D-T-P) was constructed using Cytoscape v3.7.2 software by linking the core active constituents, predicted targets, and pathways. In the network, the nodes represented the active ingredients, signaling pathways, or potential targets, while the edges identified their interactions.

### Cell viability assay

Cell viability was evaluated by MTT assay. RAW 264.7 cells were seeded in a 96-well planet and cultured overnight, followed by incubation for 12 h in BBR aqueous solution, BBR@PLGA dispersion, and BBR@PLGA@PLT dispersion containing different BBR concentrations. MTT at a final concentration of 0.5 mg/mL was added into each well and incubated at 37 °C for 120 min. The absorbance of viable cells was recorded at 560 nm by a microplate reader (Bio-Tek Instruments Inc., Winooski, VT, USA), and 750 nm was the reference wavelength. Corrected absorbance values (560–750 nm) were expressed as the percentage of viable cells relative to untreated control cells.

### Immunofluorescence staining of RAW 264.7 cells

ARG1 and CD26 immunofluorescence were performed to investigate the effect of BBR on macrophage phenotype. Ten μM BBR aqueous solution and RAW 264.7 cells were co-incubated for 12 h, followed by incubation with Anti-ARG1 Polyclonal Antibody (Solarbio, Beijing, China) and CD206 antibody (Abcam, USA). The marker expression on RAW 264.7 cells was observed by a 510 Meta-type laser scanning confocal microscopy (Carl Zeiss Microimaging Inc., Thornwood, NY) using the 488 nm and 633 nm lasers for green and red fluorescence; images were obtained at oil immersion magnifications of × 400.

### Inflammatory factors measurement

To investigate the effect of BBR and BBR@PLGA@PLT NPs on inflammatory factors secreted by macrophages, RAW 264.7 cells were treated with lipopolysaccharide (LPS) combined with corresponding drugs, and the BBR concentration in each group was 10 μM. The changes in IL-6, TNF-α, and IL-10 secreted by RAW 264.7 cells were measured by ELISA according to the kit instructions (Bioswamp, Wuhan, China).

### Rats’ MI model preparation and treatment

Male SD rats weighing 220 ± 15 g were obtained from the Experimental Animal Department of Tongji Medical College, Huazhong University of Science and Technology. The rats were anesthetized and artificially ventilated, followed by thoracotomy, and the left anterior descending coronary artery was ligated with a 6.0 suture. Fifteen minutes after the MI operation, 1.5 mL of BBR@PLGA@PLT dispersion liquid containing 0.8 mg of BBR or BBR@PLGA dispersion containing 1 mg of BBR or saline was administrated via the tail vein.

### Serum inflammatory factors measurement

The inhibitory effect of BBR@PLGA@PLT NPs on inflammation from the serum inflammatory factor levels was analyzed in rats on day three after MI. The whole blood of rats was collected, and serum was isolated. Serum levels of inflammatory factors were measured by rat IL-6 ELISA kit (Multisciences, Hangzhou, China), rat TNF-α ELISA kit (Solarbio, Beijing, China), and rat IL-10 ELISA kit (ELK biotechnology, Wuhan, China) according to the kit instructions.

### Immunofluorescence staining

All of the antibodies were obtained from Abcam (USA). Immunofluorescence staining was performed in the infarcted myocardium Sections (4 mm) on day 3 (TUNEL, CD206, and CD86) and day 28 (CX43, α-actinin, α-SMA, and CD31) post-MI. The slides were permeabilized with PBS for 35 min and blocked with 3% BSA for 35 min at 25 °C, followed by incubation with primary antibody at 4 °C for 8 h. The secondary antibody was then added and incubated for 1 h at 25 °C. DAPI was used to stain the nucleus after PBS rinsing. The percentage of positive cells was calculated by the ratio of the number of TUNEL, CD206, and CD86 positive cells to the number of nuclei. The average optical density was calculated by ImageJ to evaluate the expression of α-actinin and CX43. Loop areas that were positive for both CD31 and α-SMA were considered vessels, and the area of vascularization was calculated by ImageJ.

### Echocardiographic assessment

On the 28th day after MI, echocardiograms and left ventricular function of Rats were obtained by Philips EPIQ5 system under isoflurane anesthesia. Parasternal long-axis views at the level of the papillary muscles were obtained using transthoracic two-dimensional guided m-tracking. LVEF, FS, LVIDd, LVIDs, EDV, and ESV were calculated as the mean of 3 consecutive cardiac cycles.

### Masson staining

The collagen volume fraction was evaluated on Paraffin sections using Masson Trichrome Stain Kit (Sigma-Aldrich) according to the instructions. Collagen volume fraction was calculated as the ratio of the total area of fibrosis to the myocardium in the entire specimens and measured by ImageJ.

### Sirius red staining

Sirius red and picric acid was used for Sirius Red staining. Under polarized light, different types of collagens in sections were visualized. Red and yellow tissues were identified as collagen types I and III. ImageJ was used to quantify the collagen I/III ratio.

### Western blot

The content of TNF-α, collagen type I, and collagen type III in infarcted myocardium was assessed by Western blot. The protein concentrations were measured with BCA protein Assay Kit (Beyotime Biotechnology, China). Equal amounts of protein (20 μg) were added into each lane of a 10% SDS-PAGE (New Cell & Molecular Biotech, China) and separated. Then, the protein was transferred onto PVDF membranes (Millipore, United States). And the membranes were blocked by Protein Free Rapid Blocking Buffer (EpiZyme, China) for two h and incubated with primary antibodies (anti- TNF-α, 1:1000, Abclonal, China; anti-collagen type I, 1:1000, Proteintech, United States; anti-collagen type III, 1:5000, Proteintech, United States; anti-GAPDH, 1:1000, Abclonal, China) at 4 ℃ for 12 h. After incubation with HRP-coupled secondary antibody (goat anti-rabbit or rabbit anti-mouse IgG, 1:10,000, Boster, China), the membranes were treated with chemiluminescence (Solarbio, China) for 3 min and visualized on a Visionwork system.

### In vivo toxicity evaluation

On day 28 after MI, whole blood was collected, and serum Cr, BUN, ALT, and AST levels were measured using a biochemical analyzer (Chemray240, China). Major organs (heart, liver, lung, kidney, spleen) were harvested for H&E staining (Hematoxylin and Eosin Staining Kit, Beyotime, Shanghai, China) and TUNEL staining according to instructions.

### Statistical analysis

GraphPad Prism 9.1 (GraphPad) was used to process and analyze the data. The mean and standard deviation are presented. Statistical analyses were conducted using one-way analysis of variance analysis. *P* < 0.05 was considered statistically significant.

## Supplementary Information

**Additional file 1: Figure S1.** Ex vivo fluorescence imaging of the heart and the other major organs (liver, spleen, lung, and kidney) of post-MI rats 24 hours after Cy7-labelled BBR@PLGA@PLT NPs (a) and Cy7-labelled BBR@PLGA NPs (b) administered. **Figure S2.** Venn diagram showing the numbers of the overlapping genes between BBR and acute MI. **Figure S3.** GO enrichment analysis of the overlapping targets. **Figure S4.** “Drug-target-pathway” network: the red node is BBR, the blue nodes are target genes, the dark green nodes are herds of BBR, and the light green nodes are pathways. **Figure S5.** (a–c) Inflammatory factors are released by macrophages after different treatments. (d–f) Serum levels of inflammatory factors on day three post-MI after different treatments.

## Data Availability

Not applicable.

## Abbreviations

MI: Myocardial infarction
BBR: Berberin
PLTs: Platelets
PLGA: Polylactic-co-glycolic acid
TNF: Tumor necrosis factor
DSPE: Distearoyl phosphatidylethanolamine
IL: Interleukin
LVIDd: Left ventricular internal diameter at end-diastole
LVIDs: Left ventricular inner diameter at end-systole
ESV: End-systolic volume
EDV: End-diastolic volume
FS: Fractional shortening
LVEF: Left ventricular ejection fraction
α-SMA: α-Smooth muscle actin
Cr: Creatinine
BUN: Blood urea nitrogen
ALT: Alanine aminotransferase
AST: Aspartate aminotransferase
SD: Sprague Dawley
EDTA: Ethylenediamine tetraacetic acid
PBS: Phosphate buffer saline
