# Supplementary material for: Berberin sustained-release nanoparticles were enriched in infarcted rat myocardium and resolved inflammation
Source: J Nanobiotechnology. 2023 Jan 28;21:33. doi: 10.1186/s12951-023-01790-w (PMC9883926; doi:10.1186/s12951-023-01790-w)
Supplement: Supplementary file 1 — Additional file 1: Figure S1. Ex vivo fluorescence imaging of the heart and the other major organs (liver, spleen, lung, and kidney) of post-MI rats 24 hours after Cy7-labelled BBR@PLGA@PLT NPs (a) and Cy7-labelled BBR@PLGA NPs (b) administered. Figure S2. Venn diagram showing the numbers of the overlapping genes between BBR and acute MI. Figure S3. GO enrichment analysis of the overlapping targets. Figure S4. “Drug-target-pathway” network: the red node is BBR, the blue nodes are target genes, the dark green nodes are herds of BBR, and the light green nodes are pathways. Figure S5. (a–c) Inflammatory factors are released by macrophages after different treatments. (d–f) Serum levels of inflammatory factors on day three post-MI after different treatments. [file 12951_2023_1790_MOESM1_ESM.docx]

Additional file 1

Berberin Sustained-release Nanoparticles were Enriched in Infarcted Rat Myocardium and Resolved Inflammation

*Ke Zhu^1,2,3#^, Yu Yao^4#^, Kun Wang^1,5^, Fuqiang Shao^3^, Ziyang Zhu^1,2^, Yangmeihui Song^1,2^, Zhangyongxue Zhou^1,2^, Dawei Jiang^1,2,6^, Xiaoli Lan^1,2,6^ and Chunxia Qin*^1,2,6^*

^1^Department of Nuclear Medicine, Union Hospital, Tongji Medical College, Huazhong University of Science and Technology, No. 1277 Jiefang Ave, Wuhan, 430022, Hubei, China. ^2^Hubei Key Laboratory of Molecular Imaging, Wuhan, 430022, Hubei, China.

^3^Department of Nuclear Medicine, The First People's Hospital of Zigong, Zigong, Sichuan, China.

^4^Department of Ultrasound, The Central Hospital of Wuhan, Tongji Medical College, Huazhong University of Science and Technology, Wuhan, Hubei, China.

^5^Department of Nuclear Medicine, Shanghai East Hospital, Tongji University School of Medicine, Shanghai, China.

^6^Key Laboratory of Biological Targeted Therapy, the Ministry of Education, Wuhan, 430022, Hubei, China

**# These authors contributed equally to this work.**

***Correspondence**: qin_chunxia@hust.edu.cn

**
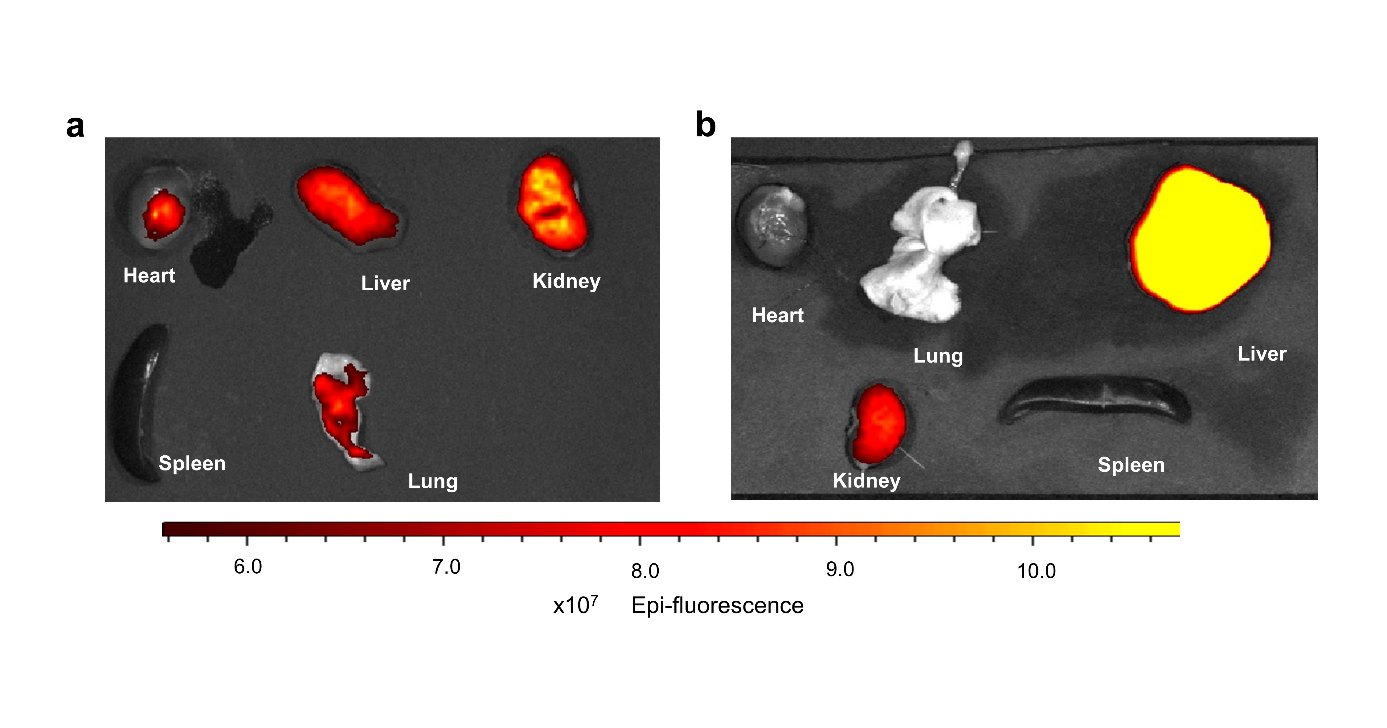
Figure S1.** Ex vivo fluorescence imaging of the heart and the other major organs (liver, spleen, lung, and kidney) of post-MI rats 24 hours after Cy7-labelled BBR@PLGA@PLT NPs (a) and Cy7-labelled BBR@PLGA NPs (b) administered.


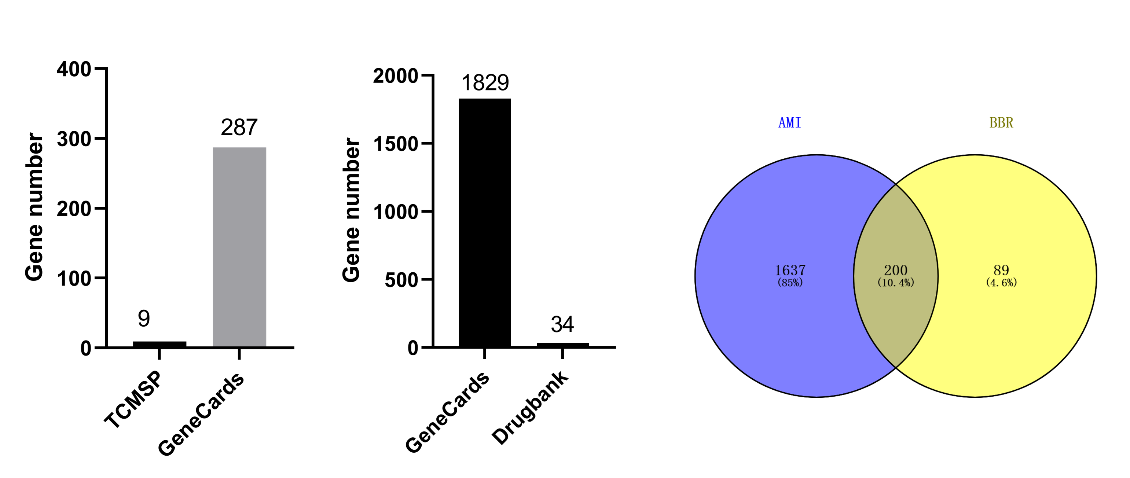


**Figure S2.** Venn diagram showing the numbers of the overlapping genes between BBR and acute MI.


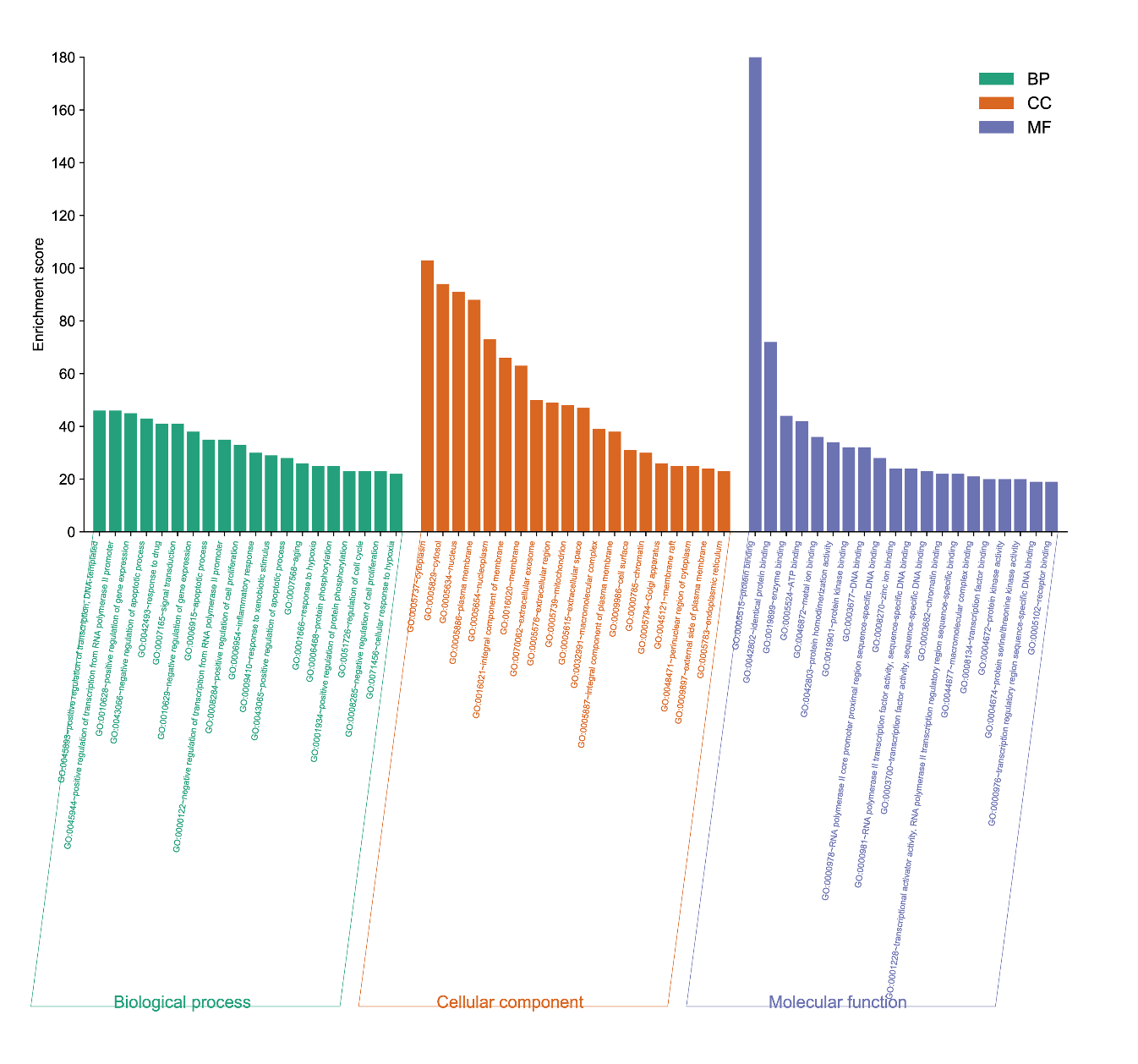


**Figure S3.** GO enrichment analysis of the overlapping targets.


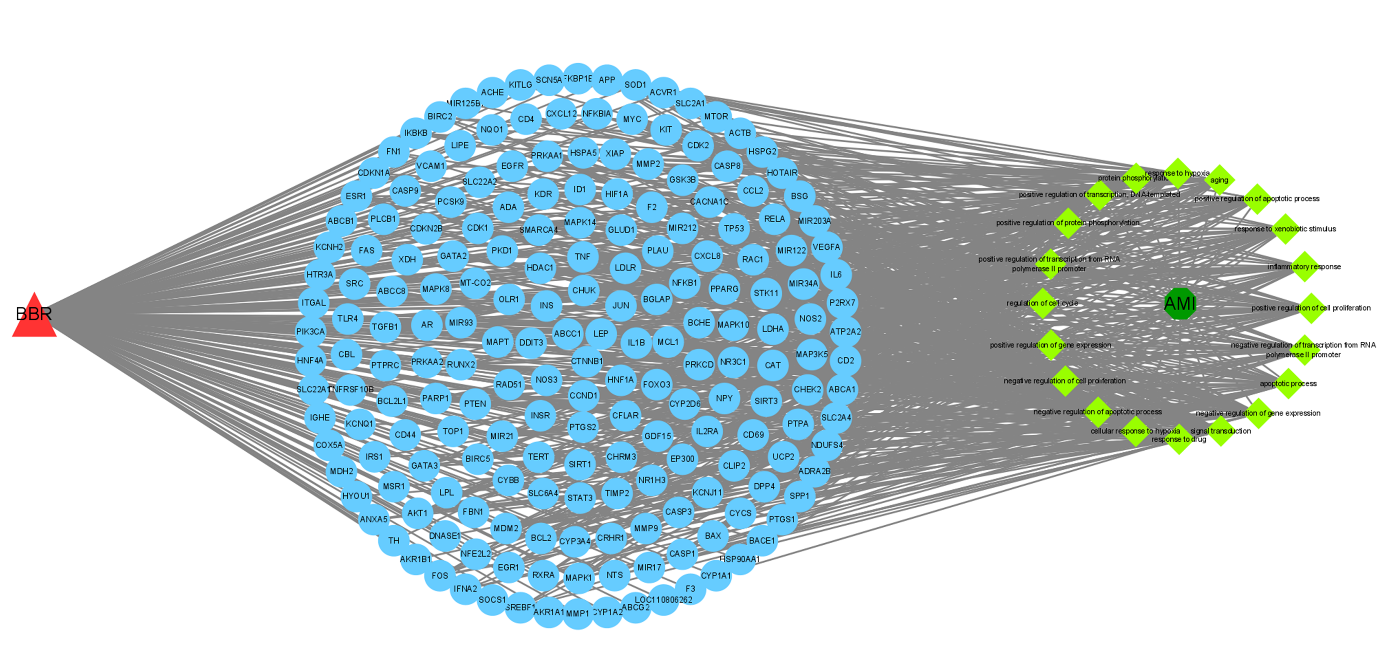


**Figure S4.**  “Drug-target-pathway” network: the red node is BBR, the blue nodes are target genes, the dark green nodes are herds of BBR, and the light green nodes are pathways.


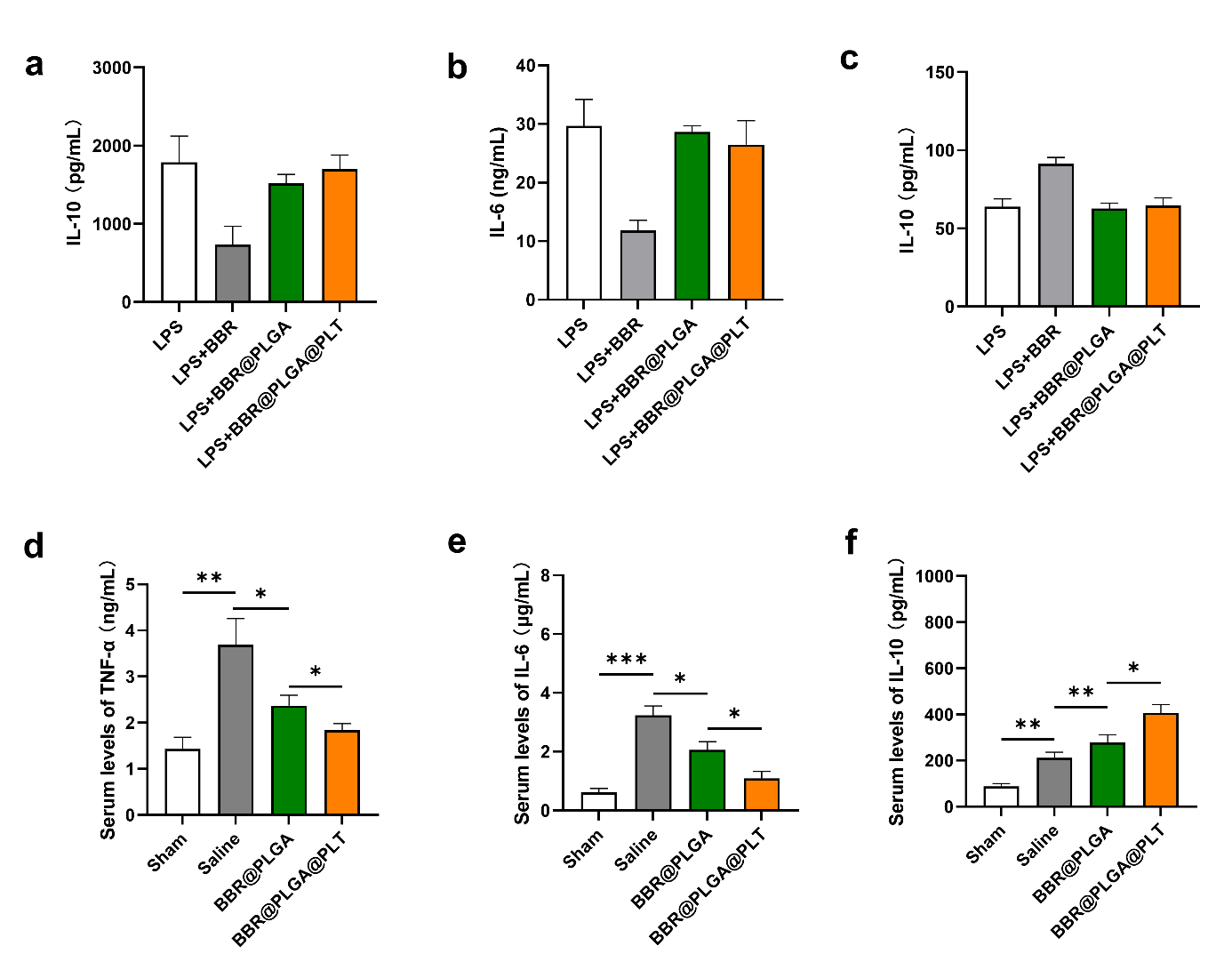


**Figure S5.** (a-c) Inflammatory factors are released by macrophages after different treatments. (d-f) Serum levels of inflammatory factors on day three post-MI after different treatments.
